# Prevalence of type 2 diabetes complications and its association with diet knowledge and skills and self‐care barriers in Tabriz, Iran: A cross‐sectional study

**DOI:** 10.1002/hsr2.1096

**Published:** 2023-02-02

**Authors:** Habib Jalilian, Elnaz Javanshir, Leila Torkzadeh, Saeedeh Fehresti, Nazanin Mir, Majid Heidari‐Jamebozorgi, Somayeh Heydari

**Affiliations:** ^1^ Department of Health Services Management, School of Health Ahvaz Jundishapur University of Medical Sciences Ahvaz Iran; ^2^ Social Determinants of Health Research Center Ahvaz Jundishapur University of Medical Sciences Ahvaz Iran; ^3^ Cardiovascular Research Centre Tabriz University of Medical Sciences Tabriz Iran; ^4^ Department of Health Policy and Management, School of Management and Medical Informatics Tabriz University of Medical Sciences Tabriz Iran; ^5^ Department of Health Economics and Management, School of Public Health Tehran University of Medical Sciences Tehran Iran; ^6^ Health Management and Economics Research Center Iran University of Medical Sciences Tehran Iran; ^7^ Department of Public Health Sirjan School of Medical Sciences Sirjan Iran

**Keywords:** comorbidity, diabetes complications, self‐care barriers, self‐management, T2DM

## Abstract

**Background and Aims:**

Diabetes can lead to multiple complications that can reduce the quality of life, impose additional costs on the healthcare systems and ultimately lead to premature death. Proper self‐care in diabetic patients can impede or delay the onset of diabetes complications. This study aimed to investigate diabetes complications and their association with diet knowledge, skills, and self‐care barriers.

**Methods:**

This was a cross‐sectional study. A total of 1139 patients with Type 2 Diabetes Mellitus (T2DM) referring to health centers in Tabriz, Iran, were included from January to July 2019. Data were collected using two questionnaires: (1) a sociodemographic questionnaire and (2) a Personal Diabetes Questionnaire (PDQ). Data were analyzed using SPSS software version 22. *χ* ^2^ test was used to examine the association between the socioeconomic and disease‐related variables and the prevalence of diabetes complications. *T*‐test was used to examine the association between diet knowledge and skills, self‐care barriers, and the incidence of diabetes complications.

**Results:**

In this study, 76.1% of patients had at least one complication, and 30.2% had a history of hospitalization due to diabetes complications during the past year. Approximately 49% and 43% were diagnosed with high blood pressure and hyperlipidemia, respectively. Cardiovascular disease was the most common diabetes complication (15.9%) and the cause of hospitalization (11.01%) in patients with diabetes. Barriers to diet adherence, blood glucose monitoring, and exercise were significantly associated with self‐reported diabetes complications (*p* < 0.001). Our results showed no significant association between the number of complications and diet knowledge and skills (*p* = 0.44).

**Conclusion:**

This study indicated that the prevalence of diabetes complications was higher among patients with more barriers to self‐care. In light of these findings, taking appropriate measures to reduce barriers to self‐care can prevent or delay the onset of diabetes complications.

## 1 INTRODUCTION

The prevalence of diabetes is rising at an alarming rate, particularly in developing nations.^1^

In 2021, 537 million adults (20–79 years) were living with diabetes. This number is projected to rise to 643 million by 2030 and 783 million by 2045. Over 3 in 4 adults with diabetes live in low‐ and middle‐income countries.^2^ According to World Health Organization (WHO), 1.5 million deaths are directly attributed to diabetes yearly.^3^ According to the latest figures from the International Diabetes Federation (IDF) in 2022, the prevalence of diabetes and total cases of diabetes in adults in Iran is estimated to be 9.5% and 5,450,300, respectively.^4^ It is projected that in Iran, 9.2 million will have diabetes by 2030.^5^

Patients with diabetes are at higher risk of morbidity, mortality,^6^ and other chronic noncommunicable or infectious diseases.^7^ Diabetes can lead to life‐threatening complications, including cardiovascular disease, retinopathy, nephropathy, neuropathy, and diabetic foot ulcer.^8^, ^9^, ^10^ However, diabetes can be controlled and managed with proper self‐care behaviors.^11^ Self‐care is an influential factor in controlling diabetes^12^ and preventing complications,^13^ and it has been shown that it can be even more effective than drug interventions.^12^

The diabetes self‐care behaviors include adherence to a dietary regime, medication adherence, regular physical activity, proper medication follow‐up, blood glucose self‐monitoring, monitoring disease progression, and foot care practices.^14^, ^15^, ^16^, ^17^ Commitment to these behaviors can reduce the risk of complications and improve quality of life.^18^, ^19^, ^20^ The ability to self‐manage depends on some factors, such as sociodemographic and clinical factors (e.g., the complexity of treatment regime and comorbidities), as well as systemic factors (e.g., social support and communication) with healthcare providers.^21^ Research also shows adequate knowledge is a significant component of diabetes management.^22^, ^23^ Diabetes knowledge is an essential precondition for effective self‐care activities and favorable health outcomes.^24^

Assessing the associations between diabetes complications and self‐care barriers and diet knowledge can help to identify barriers to self‐care that affect the incidence of diabetes complications. Assessing the associations between diabetes complications and self‐care barriers and diet knowledge can assist health policymakers and health managers in identifying the most important barriers to self‐care in diabetes complications and adopting effective measures/interventions to address the barriers in patients with T2DM. This study aimed to examine the prevalence of diabetes complications and their associations with diabetes self‐care barriers in patients with T2DM.

## 2 MATERIALS AND METHODS

### 2.1 Study design and setting

This cross‐sectional study examined the association between the prevalence of T2DM complications, diet knowledge and skills, and self‐care barriers. A cross‐sectional study helps establish preliminary evidence for a causal relationship. A total of 1139 patients with T2DM were recruited in this study. The statistical population included all patients with T2DM in Tabriz, Iran. The inclusion criteria for this study were as follows: age of ≥18 years and a confirmed diagnosis of T2DM. Those with a physical or mental disability were excluded from the study.

### 2.2 Sample size and sampling method

Using the consecutive sampling method, we included all patients referring to educational hospitals, diabetes clinics, and primary healthcare centers affiliated with Tabriz University of Medical Sciences and private endocrinologist offices from January to July 2019.

This study was a part of a PhD thesis (Grant Number; IR.TBZMED.REC.61521), and a part of this was related to the design of a questionnaire to assess reasons for forgone care in diabetic patients. Therefore, the researcher needed to perform exploratory and confirmatory factor analysis. Comrey and Lee^25^ provided the following guidance in determining the adequacy of sample size for conducting factors analysis: 100 = poor, 200 = fair, 300 = good, 500 = very good, and 1000 or more = excellent. The researchers, therefore, needed more than 1000 samples to improve sampling adequacy. However, considering the impact of sample size on factor analysis results (a larger sample will lead to more reliable results), 1200 questionnaires were distributed among subjects, and 61 questionnaires were discarded due to incomplete and/or incorrect information. Finally, 1139 patients were included in our analysis.

### 2.3 Data collection tools and data collecting process

Data were collected through two questionnaires. The first questionnaire consisted of three parts. The first part was related to sociodemographic characteristics such as age, gender, educational status, income level, and insurance coverage status. The second part was related to variables such as disease duration, Body Mass Index (BMI), current treatment type, and the history of hospital admission during the last year due to diabetes complications. The third part was questions related to comorbidities and diabetes complications such as high blood pressure status, hyperlipidemia, heart diseases, neuropathy, nephropathy, retinopathy, and foot ulcer.

The second questionnaire was the Personal Diabetes Questionnaire (PDQ). This questionnaire is a brief yet comprehensive measure of diabetes self‐care behaviors, perceptions, and barriers and is used to collect data related to the status of diet knowledge and skills, diet decision‐making, eating problems, diet adherence barriers, blood glucose monitoring barriers, medication barriers, and exercise barriers. The development and initial evaluation of the psychometric properties of the PDQ questionnaire were assessed by Stetson et al.^26^ Subscales demonstrated good internal consistency (Cronbach *α* = 0.650–0.834) and demonstrated significant associations with BMI (*p* ≤ 0.001) and HbA1c (*p* ≤ 0.001). In this study, the questionnaire was first translated from English to Persian and then back‐translated into English by a professional translator to ensure the first translation was accurate. Also, face validity was conducted by asking the endocrinologists, general practitioners, nutritionists, and public health specialists to comment on the clarity and flow of the questions in the proposed questionnaire. In our study, Cronbach's *α* was 0.81 for the total score, with subscales ranging from 0.68 to 0.84.

In our study, literate participants completed the questionnaire in 10 min, and for those who were illiterate, the questions were read to them, and they responded accordingly. Each interview lasted 20 min. Trained interviewers did all interviews.

### 2.4 Subscale description and scoring

The PDQ consists of eight subscales: diet knowledge and skills, diet decision‐making, eating problems, diet adherence barriers, blood glucose monitoring barriers, medication barriers, and exercise barriers. The diet knowledge and skills subscale comprise dietary practices concerning the type of diet information employed to direct eating behavior and consists of 9 questions. The diet decision‐making subscale is a general diet‐specific decision‐making strategy used and consists of 6 questions. The eating problems subscale focuses on behaviors that make it hard for people to lose weight and control blood sugar and consists of 3 questions. The subscales of diet adherence barriers (7 questions), blood glucose monitoring barriers (8 questions), medication barriers (8 questions), and exercise barriers (7 questions) imply environmental, social, and emotional factors interfering with attempts to adhere to the regimen. Each question is rated on a six‐point Likert scale (*Never, 1 time per month or less, 2–3 times per month, 1–2 times per week, 4–6 times per week, 1 or more times per day*). To achieve the comparability of scores of different subscales, after obtaining the raw scores of each subscale, they were converted into a standard score from 0 to 100.

The formula used to calculate the scores was as follows:

Obtainedscoreinsubscale−thepossiblelowestofsubscale/thedifferencebetweenthepossiblehighestandlowestofsubscale×100

A higher score indicates greater knowledge and skills, more frequent use of general diet‐specific decision‐making strategies, more frequent eating problems, more frequent dietary adherence, blood glucose monitoring, medication use, and exercise barriers.

### 2.5 Statistical analysis

Statistical analyses were performed using SPSS software version 22. Descriptive statistics such as frequency, mean, and standard deviation (SD) were used to examine sociodemographic and disease characteristics, the prevalence of comorbidities and diabetes complications, diet knowledge and skills, and self‐care barriers. *χ* ^2^ test was used to examine the association between the socioeconomic and disease‐related variables and the prevalence of diabetes complications. *T*‐test was used to examine the association between diet knowledge and skills, self‐care barriers, and the incidence of diabetes complications. A generalized linear model regression was used to assess the factors influencing the number of diabetes complications. Multivariate linear regression was applied to assess the effect of self‐care barriers on the number of diabetes complications. The tests were carried out at a 5% significance level, and a *p* ≤ 0.05 was considered significant.

### Ethics approval and consent to participate

This study was a part of a comprehensive PhD thesis work, ethically approved by the Ethics Committee of Tabriz University of Medical Sciences (Reference Number; IR.TBZMED.REC.1397.166). All participants were assured that the data would be confidential and anonymous. Verbal informed consent was obtained from all participants involved in this study. Informed consent from all participants has been obtained. All methods were performed in accordance with relevant guidelines and regulations that must be considered in research where humans are involved.

## 3 RESULTS

The sociodemographic and disease characteristics of the 1139 participants are shown in Table 1. The mean age of participants was 56.93 ± 13.34. Two‐thirds of the participants were women, and most (41.5%) were illiterate. The yearly household income of 74.8% was >2287.26 (PPP, Current International $). More than two‐thirds of participants were not covered by supplemental insurance. Most of the patients (88.1%) resided in urban areas. Most participants were on oral medicine, and just 6.2% changed their lifestyle as the main treatment strategy. The mean duration of diabetes and the mean BMI of the participant were estimated at 9.06 ± 7.12 years and 28.37 ± 5.27, respectively.

**Table 1:** The prevalence rate of diabetes complications is based on demographic and socioeconomic, and disease characteristics.

Variable	Modes	Frequency (%)	Complications %	*ϰ* ^2^	*p* Value
Gender	Male	384 (33.7)	72.7	3.82	0.05
	Female	755 (66.3)	77.9		
Age	<40	127 (11.2)	43.3	99.21	<0.0001*
	40–60	527 (46.3)	75.3		
	>60	485 (42.6)	85.6		
Income status^a^	$<2287.26	397 (52.5)	74.8	0.32	0.56
	$>2287.26	359 (47.5)	73.0		
Education status	Illiterate	473 (41.5)	86.7	66.06	<0.0001*
	Reading and writing ability	407 (35.7)	73.5		
	Diploma	195 (17.1)	63.6		
	Academic education	64 (5.6)	53.1		
Type of basic health insurance	Social security	707 (64.4)	75.8	0.02	0.88
	Iranian health insurance	391 (35.6)	76.2		
Supplementary health insurance status	Yes	430 (39.1)	78.2	1.72	0.19
	No	669 (60.9)	74.8		
Habitation status	Rural area	135 (11.9)	75.6	1.25	0.25
	Urban area	1003 (88.1)	80.0		
Disease duration	<5 year	421 (37.1)	64.8	50.11	<0.0001*
	5 to 10 years	260 (22.9)	78.5		
	>10 years	455 (40.1)	85.1		
Type of current treatment	Oral pills	619 (54.3)	76.1	23.17	<0.0001*
	Insulin	449 (39.4)	79.7		
	Change in lifestyle	71 (6.2)	53.5		
Body mass index	Normal weight	302 (26.9)	66.9	20.17	<0.0001*
	Overweight	445 (39.7)	78.7		
	Obese	374 (33.4)	80.7		
	No	795 (69.8)	70.2		
The history of forgone care during last year	Yes	510 (44.8)	79.6	6.64	0.01*
	No	623 (54.7)	73.0		

^a^ (PPP, Current International $), 2020 (https://data.worldbank.org/indicator/NY.GDP.PCAP.PP.CD).

* *p* < 0.05 was considered significant.

As shown in Table 1, the variables of gender, age, education, BMI, disease duration, type of treatment, and history of forgoing treatment were significantly associated with the presence of diabetes complications (*p* < 0.05). Women, older people, those with lower education, higher BMI groups and those with longer disease durations, those with a history of forgoing treatment, and those whose main treatment was based on insulin injection were more likely to have diabetes complications. Moreover, the results of the Pearson's correlation showed that the number of complications was significantly positively correlated with age (CC = 0.31, *p* < 0.0001), disease duration (CC = 0.29, *p* < 0.0001), and BMI (CC = 0.16, *p* < 0.0001).

**Figure 1:**
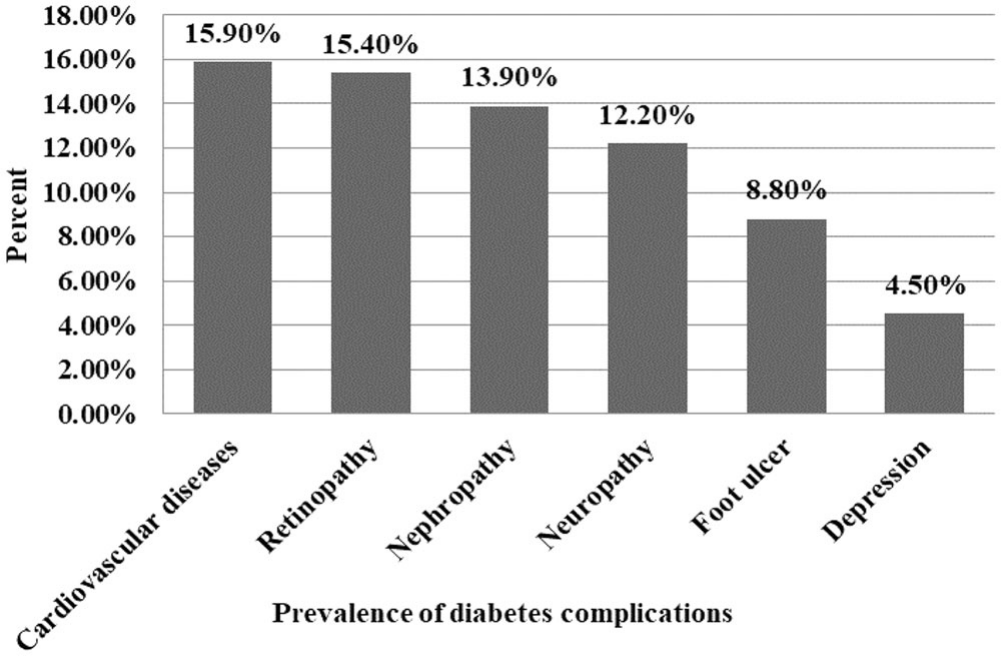
The prevalence of diabetes complications.

**Figure 2:**
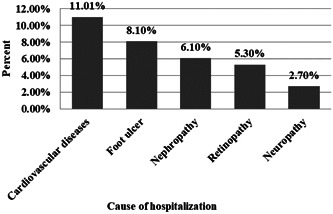
The most common cause of hospitalization in patients with T2DM.

**Figure 3:**
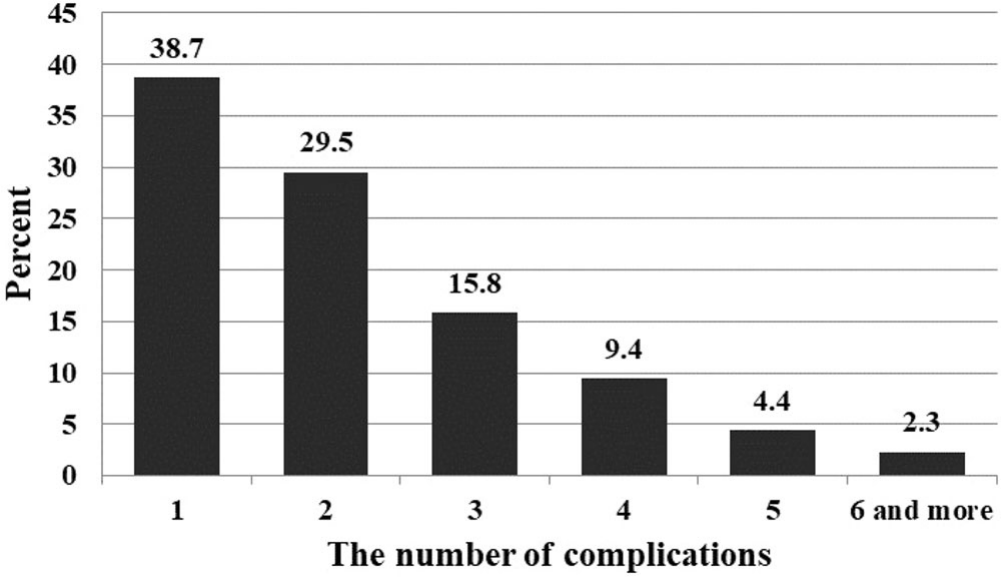
The percentage of patients based on the number of self‐reported complications.

Results showed that 76.1% of patients had at least one complication. In our study, 48.6% and 42.7% were diagnosed with high blood pressure and hyperlipidemia, respectively. Figure 1 shows the percentage of the prevalence of diabetes complications. The most common complications of diabetes were cardiovascular disease (15.9%) and retinopathy (15.4%). As shown in Figure 2, 30.2% of patients had a history of hospitalization due to complications of diabetes during the past year, and cardiovascular disease (11.1%) was the most common cause of hospitalization. Figure 3 presents the percentage of patients based on the number of self‐reported complications. Most patients (38.7%) had at least one diabetes complication.

The mean score of reported barriers to diabetes self‐care in individuals with and without complications is presented in Table 2. The most frequently reported categories in both groups were diet barriers, exercise barriers, and blood glucose monitoring barriers. Barriers to diet adherence, blood glucose monitoring, and exercise were significantly associated with self‐reported T2DM complications (*p* < 0.05). Diabetes complications were more prevalent in patients with more diet adherence, monitoring, and exercise barriers. Moreover, the results of the Pearson correlation showed a significant and positive correlation between the number of complications and diet barriers (CC = 0.08, *p* = 0.003), blood glucose monitoring barriers (CC = 0.06, *p* = 0.02), as well as exercise barriers (CC = 0.23, *p* < 0.0001).

**Table 2:** The mean of reported self‐care barriers and its association with diabetes complications.

Variable	With complications	Without complications	*t*	*p* Value
Diet knowledge and Skills	45.31 (23.51)	44.62 (25.85)	0.39	0.69
Eating problems	19.06 (21.56)	19.36 (20.10)	−0.21	0.83
Diet barriers	24.12 (17.56)	21.09 (17.07)	2.53	0.01*
Medication barriers	12.53 (17.40)	12.91 (15.04)	−0.34	0.73
Exercise barriers	22.37 (17.61)	15.80 (15.01)	6.01	<0.0001*
Blood glucose monitoring barriers	17.26 (16.97)	14.82 (14.64)	2.27	0.02*
Diet decision making	39.81 (23.09)	38.19 (25.02)	0.94	0.34

* *p* < 0.05 was considered significant.

The results of the generalized linear model regression are represented in Table 3. The omnibus test results (*p* < 0.05) indicate that the current model outperforms the null model. Older age, obesity, longer disease duration, lower education level, having a history of forgone care, and exercise barriers were significantly positively associated with diabetes complications.

**Table 3:** Generalized linear regression model for diabetes complications.

Parameter	*B*	95% Wald CI		Hypothesis test	
		Lower	Upper	Wald *χ* ^2^	*p* Value
(Intercept)	1.155	0.503	1.807	12.051	0.001*
Age (reference = 60 years)					
<40 years	−0.601	−0.965	−0.236	10.436	0.001*
40–60 years	−0.332	−0.547	−0.117	9.155	0.002*
Gender (reference = female)					
Male	0.089	−0.119	0.298	0.710	0.40
Marital status (reference = married)					
Single	0.205	−0.043	0.453	2.618	0.10
Habitant status (reference = rural)					
Urban	−0.095	−0.411	0.222	0.342	0.55
Income status (reference = 30 million Rials)					
<30 million Rials	−0.039	−0.261	0.183	0.118	0.73
Education status (reference = academic education)					
Illiterate	0.668	0.239	1.096	9.310	0.002*
Reading and writing ability	0.432	0.044	0.821	4.758	0.02*
Diploma	0.217	−0.176	0.610	1.170	0.27
Type of basic health insurance (reference = Iranian health insurance)					
Social security	0.065	−0.137	0.266	0.398	0.52
Supplementary health insurance coverage (reference = no)					
Yes	0.095	−0.117	0.306	0.768	0.38
BMI (reference = obese)					
Normal weight	−0.272	−0.520	−0.025	4.668	0.03*
Overweight	−0.145	−0.366	0.075	1.677	0.19
Disease duration (reference = 10 years)					
<5 years	−0.372	−0.608	−0.136	9.563	0.002*
5 to 10 years	−0.223	−0.474	0.027	3.047	0.08
Type of current treatment (reference = insulin)					
Change in lifestyle	−0.598	−1.026	−0.169	7.483	0.006*
Oral pills	−0.172	−0.385	0.041	2.518	0.113
Forgone care (reference = no)					
Yes	0.361	0.168	0.554	13.451	<0.0001*
Diet knowledge and skills	−0.002	−0.007	0.003	0.421	0.51
Eating problems	−0.001	−0.006	0.004	0.046	0.83
Diet barriers	0.003	−0.004	0.010	0.641	0.42
Medication barriers	−0.007	−0.014	0.0001	3.545	0.06
Blood glucose monitoring barriers	0.001	−0.007	0.008	0.019	0.89
Exercise barriers	0.016	0.009	0.023	19.720	<0.0001*
Decision‐making diet	0.001	−0.004	0.006	0.175	0.67
(Scale)	1.423	1.279	1.584		

* *p* < 0.05 was considered significant.

Multivariate linear regression was used to control for potentially confounding variables. According to the results, after controlling for age, gender, marital status, habitant status, income, education, insurance status, BMI, disease duration, treatment regime and history of forgone care, diet, blood glucose monitoring, and exercise barriers were positively associated with the number of diabetes complications (Table 4).

**Table 4:** Multivariate linear regression model for T2DM complications.

Variable	Beta In	*T*	*p* Value
Diet knowledge and skills	−0.027	−0.765	0.44
Eating problems	0.031	0.843	0.40
Diet barriers	0.091	2.492	0.01*
Medication barriers	0.030	0.821	0.41
Blood glucose monitoring barriers	0.075	2.045	0.04*
Exercise barriers	0.179	4.973	<0.0001*
Decision‐making diet	−0.023	−0.641	0.52

* *p* < 0.05 was considered significant.

## 4 DISCUSSION

This study aimed to investigate the prevalence of T2DM complications and their association with self‐care barriers. We found more than two‐thirds of patients had at least one complication, and 30.2% of patients had a history of hospitalization due to diabetes complications during the past year. Also, 48.6% and 42.7% of patients, respectively, had high blood pressure and hyperlipidemia, indicating the poor performance of Iran's health system in diabetes management and control. The high rates of complications among participants may be due to various factors, such as late diagnoses of diabetes, late initiation of treatment, poor adherence to treatment regimens, lack of access to therapy, poor self‐care, and even lifestyle factors. Therefore, given the high prevalence of diabetes complications, it seems that preventive care strategies and self‐care measures, in addition to pharmacologic treatments to control diabetes complications, should be taken into account effectively.

In our study, CVD was the most prevalent diabetes complication. Also, CVD was the most common cause of hospitalization in patients with diabetes. Previous studies demonstrated that CVD is the most common adverse outcome of diabetes,^27^ and people with diabetes have about twice the risk of CVD compared to those without.^28^ In a study in Iran in 2016, 56.60% of men and 56.20% of women had at least one chronic vascular complication of diabetes.^29^ In a study conducted by Kosiborod et al.^30^ in 38 countries, approximately 80% of patients with T2DM develop cardiovascular complications, which account for approximately 65% of deaths in this group. In Iran's primary healthcare system, patients with diabetes are visited monthly by primary healthcare providers and every 3 months by general physicians. Given the high prevalence of cardiovascular diseases and the socioeconomic burden of the disease in patients with T2MD, it is suggested that patients with diabetes are visited at least every 6 months by a cardiologist and checked for cardiovascular complications.

The American Heart Association (AHA) and American Diabetes Association (ADA) recommend intensive management of cardiovascular risk factors in patients with diabetes.^31^ The use of statin, in addition to intensive blood pressure management for these patients, can be useful, according to the European Society of Cardiology guidelines (ESC).^32^ Research has shown that better glycemic control, blood pressure management, glucose‐lowering agents, and pharmacologic interventions can significantly reduce the risk of cardiovascular disease among T2DM.^33^, ^34^, ^35^, ^36^ It is interesting to note that the application of the 5 A's self‐management (assess, advise, agree, assist, and arrange) support model can have a considerable impact on the improvement of preventing cardiovascular complications behaviors (PCCB) in elderly patients with T2DM.^37^

In this study, the odds of having diabetes complications were higher in women, older patients, and those with lower education. This can be attributed to factors such as limited access to healthcare and poor adherence to self‐care due to socioeconomic problems. Owing to the effect of demographic and socioeconomic factors on the incidence of complications, interventions/measures for managing and controlling the disease should not be the same for all patients. Therefore, to effectively manage the disease and reduce the prevalence rate of diabetes complications, it is necessary to modulate patient care based on different groups and the prevalence rate of complications and perform follow‐up care at shorter intervals for patients with diabetes, particularly high‐risk patients. Several studies have demonstrated that a higher educational level^38^, ^39^, ^40^ contributes to patients' ability to care for their diabetes. Azreena et al.^41^ stated that adequate health literacy is a prerequisite for effectively utilizing the measures for efficient diabetes management. Regarding gender differences, a study reported that women with T2D are less likely to adhere to their drug treatment regimen than men.^42^

Our results showed that diabetes complications were positively and significantly correlated with disease duration. It appears that disease management interventions such as patient education and self‐care measures to reduce or delay complications should be initiated from the early stages of the disease. Seung and colleagues demonstrated that a structured diabetes education program's effectiveness could be affected by diabetes duration. They suggest that people with T2DM who had experienced a longer diabetes duration before participating in diabetes education showed lower adherence to physical activity frequency and dietary habits.^43^

We also found that those with a history of forgone care during the last year were more likely to have diabetes complications. Forgone care can be attributed to some factors, such as access barriers to healthcare, low quality of services provided by health providers, and unaffordability of the treatment costs for diabetes patients. Patients with diabetes require comprehensive and continuous medical care. Hence, for effective management of diabetes complications, it is necessary to identify and address the potential reasons for forgone care. A study by Jalilian et al.^44^ demonstrated that the most important reason for forgoing care in patients with T2DM was financial barriers resulting from treatment costs.

In this study, those who were obese were more likely to have diabetes complications. This result highlights the need for lifestyle interventions and self‐care measures such as diet and exercise for complication management. These interventions must be carried out more intensively in obese and overweight patients. Our findings are in keeping with the results of Omar et al.^45^ which indicated obesity is a major risk factor for many NCDs and their complications, including T2DM, CVD, hypertension, and stroke.

According to the multivariate linear regression results and after controlling the effect of socioeconomic and underlying variables, the results confirmed that diet, blood glucose monitoring, and exercise barriers had an intensification effect on the incidence of diabetes complications. Our findings are consistent with a previous study that reported physical activity barriers were significantly associated with self‐reported complications.^46^ These barriers should be identified in individuals of different groups, and appropriate measures should be taken to remove the barriers among vulnerable groups to prevent and delay complications.

Regarding diet, some studies reported that patients' lack of knowledge of a specific diet plan and perceived belief in the social unacceptability of healthy behaviors hindered healthy eating and physical exercise participation.^47^, ^48^ Since the score of diet knowledge and skills was less than moderate, one way of improving diet self‐care can be enhancing the nutritional literacy of patients through education about diet and strengthening the role of a nutrition consultant in the treatment team. A study showed that people with higher health literacy were more empowered and those with higher empowerment were more likely to eat healthy foods and exercise.^49^ A study by Sami and colleagues demonstrated a significant positive association between knowledge of diabetic diet and dietary practices. They also reported knowledge is a salient factor related to dietary behavior control.^50^ Previous Studies also demonstrated that nutrition education could improve diabetes‐related nutrition knowledge and dietary practices in diabetic patients.^51^, ^52^ Didarloo et al.^53^ reported diabetes knowledge (including diet‐related) is significantly correlated to better psychosocial self‐efficacy and a strong predictor of behavioral intention and dietary behaviors in diabetic patients.

Furthermore, in addition to a lack of dietary knowledge, financial constraints make patients unable to buy healthy food.^54^ Sarpooshi et al.^55^ reported that access to fruit and nutrition in the diabetic diet in Iran was directly related to the patient's economic status. At the micro level, the government can provide nutritional support packages for patients with a low economic status. At the macro level, food subsidy programs, as a strategy to promote healthy nutrition, can reduce socioeconomic inequalities in health. Also, the government can impose a tax on unhealthy food.

In our study, the main barriers to diet and exercise self‐care adherence were depression, frustration, high workload, and family problems. Depression among those with T2DM is up to twice as common as those without.^56^ Healthcare providers should promote self‐care management education among these patients, as self‐management education can reduce psychological factors such as depression and distress and improve diabetes control and the overall quality of life in patients with T2DM.^57^ A study revealed that diabetic patients receiving more social support from their family and friends are more successful in adherence to self‐care behaviors.^58^

In this study, most patients reported that they could not afford to buy blood glucose monitoring devices. The government can allocate funding for continuous glucose monitoring for people with diabetes, and the costs should be reimbursed by the government and insurance companies. A study reported that the most common barriers to continuous glucose monitoring use were the high cost of the device, lack of insurance coverage, the hassle of wearing devices, and the dislike of having devices on the body.^59^ Ong et al.^60^ showed that the factors that influenced the self‐monitoring of blood glucose were mainly related to cost, participants' emotions, and the self‐monitoring of the blood glucose process. In the United States, reimbursement criteria have been announced for therapeutic continuous glucose monitoring devices for patients with T1DM and T2DM on intensive insulin treatment.^61^ In many European countries, continuous glucose monitoring expenses are now covered by national healthcare systems, thus increasing the accessibility of such technology.^62^

### 4.1 Limitations and strengths

This study comprehensively investigated the relationship between demographic, socioeconomic, and disease characteristics variables and self‐care barriers and the incidence of diabetes complications. However, this study has some limitations. First, barriers related to the healthcare provider/system were not assessed to carry out self‐care. Second, because this is a cross‐sectional study, we cannot determine for certain whether self‐care barriers lead to the incidence of complications or conversely. Finally, since the assessment of diabetes complications was based on patients' self‐reported information, the prevalence of complications may be underestimated. Therefore, it is suggested that the prevalence of complications be studied through patients' records to achieve more accurate results.

### 4.2 Policy implications

Effective management to prevent or delay the development of diabetes and diabetes‐related complications is a complex process requiring appropriate self‐care measures and pharmacologic treatments. According to our results, the likelihood of the incidence of diabetes complications was higher in patients who had more barriers to self‐care. Reducing or addressing barriers to self‐care, such as diet adherence and physical activity, requires improving patients' socioeconomic status. This calls for a holistic collaboration between the health system and all relevant institutions and organizations. Moreover, considering that the prevalence of diabetes complications differed depending on age and gender groups, subjects with different education levels, disease duration, and BMI, appropriate interventions to prevent or delay diabetes complications should be adopted for different groups, with a particular emphasis on vulnerable groups. Hence, health policymakers and healthcare providers need to take into consideration these factors when designing and implementing preventive interventions.

## 5 CONCLUSIONS

This study showed that the prevalence of complications of diabetes was considerable and was higher in those with more self‐care barriers. Therefore, taking appropriate measures to reduce self‐care barriers can lead to better self‐care adherence and ultimately delay and reduce diabetes complications. In our study, cardiovascular disease was found to be the most common diabetes complication and the cause of hospitalization in patients with diabetes. Controlling comorbidities such as hypertension and targeting strategies to promote vascular health can be paramount in reducing the cardiovascular complications of diabetes.

## AUTHOR CONTRIBUTIONS

**Habib Jalilian**: Conceptualization; data curation; formal analysis; methodology; resources; software; supervision; validation; writing – original draft; writing – review & editing. **Elnaz Javanshir**: Writing – original draft. **Leila Torkzadeh**: Writing – original draft. **Saeedeh Fehresti**: Writing – original draft. **Nazanin Mir**: Writing – original draft. **Majid Heidari‐Jamebozorgi**: Investigation. **Somayeh Heydari**: Writing – original draft; writing – review & editing.

## CONFLICT OF INTEREST STATEMENT

The authors declare no conflict of interest.

## TRANSPARENCY STATEMENT

The lead author Habib Jalilian affirms that this manuscript is an honest, accurate, and transparent account of the study being reported; that no important aspects of the study have been omitted; and that any discrepancies from the study as planned (and, if relevant, registered) have been explained.

## Data Availability

The data sets used and/or analyzed during this study are available from the corresponding author upon reasonable request.
